# Service user views of spiritual and pastoral care (chaplaincy) in NHS mental health services: a co-produced constructivist grounded theory investigation

**DOI:** 10.1186/s12888-016-0903-9

**Published:** 2016-06-17

**Authors:** Julian Raffay, Emily Wood, Andrew Todd

**Affiliations:** Spiritual and Pastoral Care, Mersey Care NHS Foundation Trust, Indigo Building, Ashworth Hospital Parkbourn, Liverpool, L31 1HW England; Cardiff Centre of Chaplaincy Studies, St Michael’s College, 54 Cardiff Road, Llandaff, Cardiff CF5 2YJ Wales

**Keywords:** Chaplaincy, Co-production, Spiritual and pastoral care, Service user perspectives, Participation, Grounded theory, Qualitative research

## Abstract

**Background:**

Within the UK National Health Service (NHS), Spiritual and Pastoral Care (SPC) Services (chaplaincies) have not traditionally embraced research due to the intangible nature of their work. However, small teams like SPC can lead the way towards services across the NHS becoming patient- centred and patient-led. Using co-production principles within research can ensure it, and the resulting services, are truly patient-led.

**Methods:**

A series of interviews were conducted with service users across directorates of a large NHS mental health Trust. Their views on the quality of SPC services and desired changes were elicited. Grounded theory was used with a constant comparative approach to the interviews and analysis.

**Results:**

Initial analysis explored views on spirituality and religion in health. Participants’ concerns included what chaplains should do, who they should see, and how soon after admission. Theoretical analysis suggested incorporating an overarching spiritual element into the bio-psycho-social model of mental healthcare.

**Conclusions:**

Service users’ spirituality should not be sidelined. To service users with strong spiritual beliefs, supporting their spiritual resilience is central to their care and well-being. Failure will lead to non-holistic care unlikely to engage or motivate.

## Background

Public spending is under intense scrutiny. NHS services need to justify their funding. Spiritual and Pastoral Care (SPC) services (also called chaplaincies) have traditionally stayed away from standard outcome measures as they do not fit with the ethos of the service. This must change as organisations including the National Secular Society have campaigned to have NHS funding removed from SPC [1]. If SPC is to survive and modernise, research and outcome measures are unavoidable [2]. Developing suitable and reliable measurement within the field is vital.

SPC departments have traditionally lacked other NHS departments’ protocols or guidance. Recent guidelines [3, 4] have been more about recommended staffing numbers and training than the day-to-day activities conducted by chaplains. The impact of such voluntary competencies is unclear [5]. The lack of clarity about what chaplains should be doing makes outcome measures difficult to design [2].

Chaplains have been likened to advocates, providing cultural advice and support [6] but they also support spiritual and religious observance. A collaborative (as opposed to a dependent) religious coping style (working with God rather than waiting for God to fix things) correlates with a positive impact on mental health and recovery [7, 8]. Table 1 presents the working definitions of key words used in this paper, recognising the literature has not reached consensus and concepts overlap.

**Table 1:** Key definitions: Chaplaincy, spirituality, religion, pastoral, and resilience

Term	Definition
Chaplaincy	Modern healthcare chaplaincy is a service and profession working within the NHS that is focused on ensuring that all people, be they religious or not, have the opportunity to access pastoral, spiritual or religious support when they need it [4]
Spirituality	A phenomenon unique to the individual and has been defined as the “breath” that animates life or a sense of connection to oneself, others, and that which is beyond self and others, spirituality is an individual construct, denoting a personal relationship with the transcendent [42]
Religion	Religion is an organised system of beliefs, practices, rituals and symbols designed a) to facilitate closeness to the sacred or transcendent (God, higher power, or ultimate truth/reality) and b) to foster an understanding of one’s relationship and responsibility to others in living together in a community. [43]
Pastoral care	Pastoral care is rooted in non-judgemental listening and attentiveness to service-users, carers and staff. It pays supportive and enabling attention to a range of human needs and aspirations, in the context of healthcare, being especially alert to questions of identity and belief (whether presented as religious, spiritual or neither of those).
Resilience	Resilience is the ability of an individual to respond to stress in a way that is healthy and adaptive and allows personal goals to be achieved with the minimum psychological and physical cost [44]

Chaplains can work with service users and carers to build resilience. Resilience and spirituality have numerous links including finding meaning in life and having a sense of hope [9]. These concepts also overlap with the Recovery Model’s recovery processes: connectedness, hope, identity, meaning in life and empowerment (CHIME) [10] which have a spiritual component. Although Leamy and colleagues associated spirituality with meaning in life [10], there may be greater association. Many people experience connectedness to others of faith, to humanity, nature, or the Universe as a whole as part of their spirituality.

Co-designing and co-evaluating services can make them truly patient-centred. Co-production recognises that everyone has a vital contribution to make and brings people who use mental health services, carers, and staff together on equal terms [11]. It creates opportunities to understand each other’s concerns and builds on recovery approaches by facilitating empowerment [12]. In this research, the research team comprising service users, carers, and staff, explored what service users value in their spiritual and pastoral care and what changes they want.

## Method

Co-production is a key philosophy of the research team. The patient and public involvement panel (Panel) was recruited from the start and contributed throughout. It comprised NHS service users and carers. Contributions (in keeping with INVOLVE recommendations [13]) included writing the interview schedule, piloting interviews, deciding which service user groups to target, and insights for the analysis [14]. To explore service user perceptions, grounded theory with its origins in the symbolic interactionist approach of Mead offered the most promising approach [15]. Constructivist grounded theory was chosen as the researchers were already immersed in the participant’s context prior to the study [16]. Comparing interviews using the constant comparative method allowed deep penetration into the lived experience of mental health service users on psychiatric wards. This was in part based on Kara’s insight that research team members may hold multiple roles and 'mutable identities' [17]. Interpreting findings with the Panel ensured fidelity to the data and mitigated the impact of the researchers’ predetermined expectations [18].

Semi-structured interviews were conducted at a place and time convenient to participants. Potential participants were given at least 24 hours to consider joining the research. Before the interview commenced, participants were told the research’s purpose and aims. They received guidance about how they could withdraw consent at any point. All participants had capacity to consent and gave informed consent to be in the research and for their data to be used in the write up. Topics that appeared important to earlier participants were included in later interviews to elaborate on the issues. Thus interview schedules were altered in keeping with the constant comparative method [18]. Theoretical saturation was considered to have been reached.

Audio recorded interviews with participants were undertaken by one of two researchers (JR and EW). EW transcribed verbatim, checking for accuracy, and removing names and identifiers. Participants were invited to review their transcripts. Four asked to do this without reporting errors. To support reflective practice and enable constant comparison [19], transcriptions, coding, and analysis were completed as soon as possible after each interview.

Initial line-by-line coding was completed by EW, to explore meanings and actions, but remaining close to the data [19]. Focused coding re-evaluated the initial codes, combining some before grouping codes into categories. JR and AT cross-checked the coding and analysis. The whole team had extensive discussions in person and by email to decide on the final categories. The final step involved conceptualising what had been said and generating a theory grounded in the data [20]. The Panel provided feedback on the results. The data was managed using NVivo software.

### Reflexivity

The researchers are a Christian chaplain (JR), and an academic (AT) with constructivist backgrounds, and a mental health nurse (EW) with a critical realist background, identifying as ‘spiritual but not religious’. This diversity allowed the team to challenge assumptions and discuss preconceptions. Some of the participants knew JR in his capacity as chaplain prior to the commencement of interviews. EW was new to the Trust and did not establish a relationship with participants prior to the interviews. AT had no direct contact with participants (other than meeting the Panel). Participants were informed of the research team’s motivation for studying this area but not the motivations of individuals. Information about individual researchers was limited to their name, professional background, and how to make contact later if they wished to complain/comment further. Service user volunteers from Mersey Care NHS Foundation Trust’s adult acute, medium and high secure services were recruited, mainly by a chaplain attending routine ward meetings. These wards were chosen to reflect a variety of inpatient experience, SPC use, treatment, and demographics. Theoretical sampling was attempted within each ward but reliance on psychiatric inpatient volunteers and a small population curtailed the possibilities. Mersey Care covers the North Merseyside region of North West England. According to the 2011 census, Merseyside is more religious, more Christian and more socially and economically deprived than the UK average [21, 22].

Five pilot interviews were undertaken in January and February 2015 to ensure processes were safe for participants and researchers alike and valid for the purposes of the research. Pilot participants came from the Panel. They recommended changes to the wording of the standard consent form around access to patient records. This was resubmitted and the ethics committee approved the revision.

Between April 2015 and August 2015, a further seventeen service users were interviewed in private rooms on the participant’s ward or unit. In most cases only the participant and interviewer were present. For two interviews a student nurse observed; explicit consent was sought for this. Participants were only interviewed once; interviews lasted from seven minutes to one hour. The participant demographics are shown in Table 2. The age data is incomplete due to missing data.

**Table 2:** Participant demographics

Demographic	Type	Number of participants
Gender	Male	17
	Female	5
Age	Under 40	5
	40–59	8
	60 and over	6
Relationship to the Trust	Open acute ward service user	10
	Secure^ ward service user	7
	Community service user	2
	Carer	3
Faith group	Atheist	1
	Did not identify	2
	Multiple	1
	Christian (no denomination)	1
	Church of England	5
	Roman Catholic	8
	Pentecostal	2
	United Reform	1
	Born-Again	1

^Secure in this context refers to high and medium secure mental health units. It does not include psychiatric intensive care (PICU), low secure units, or prison inreach services

## Results

### Categories

**Fig. 1:**
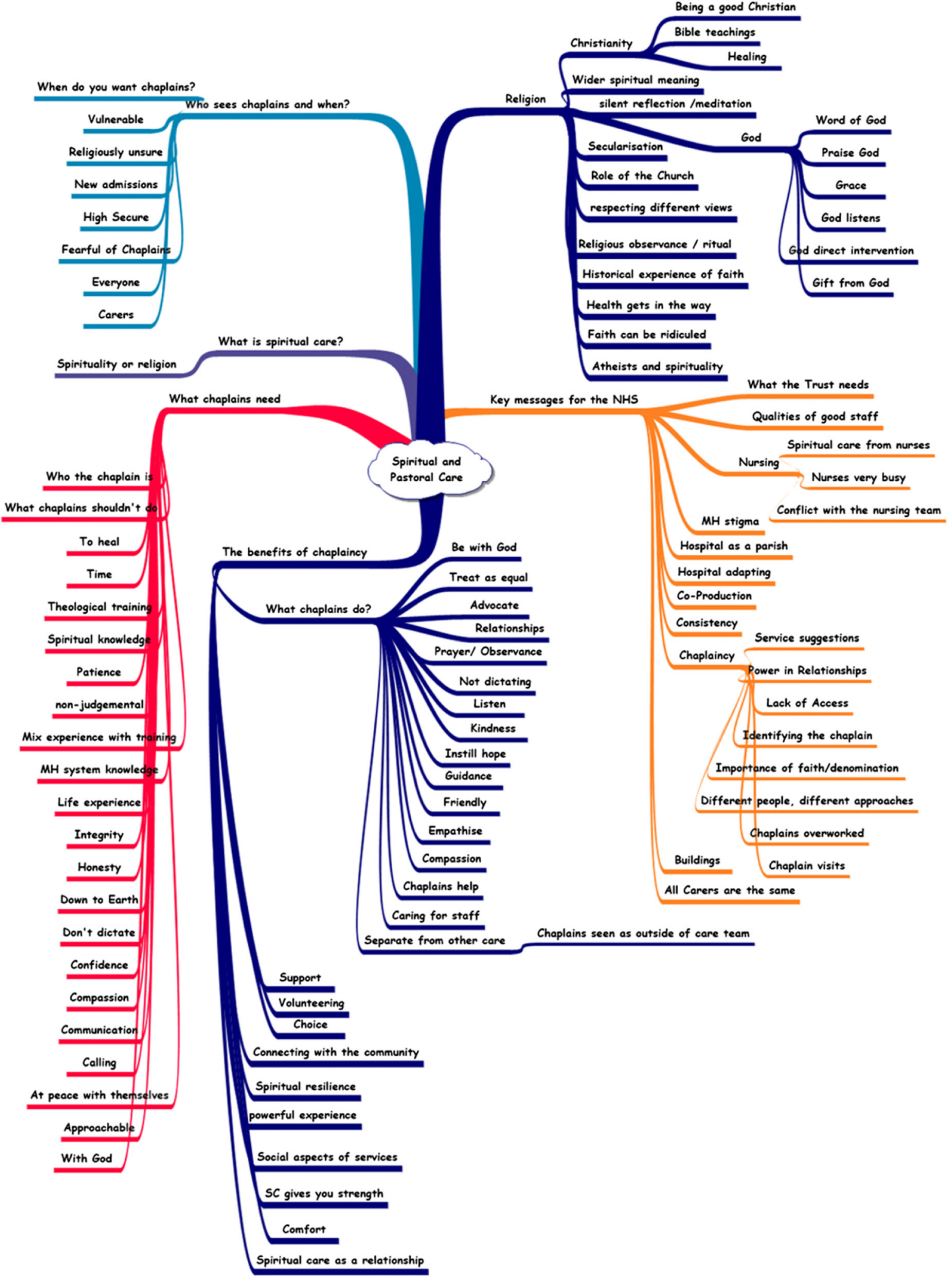
A category map illustrating the breadth of views elicited in the interviews

Six categories emerged from the interviews. These were: (1) the meaning of spiritual care, (2) benefits of the SPC department, (3) the role of religion, (4) qualities of a ‘good’ chaplain, (5) who talks to chaplains and when, and (6) chaplains and the multidisciplinary team. The category map (Fig. 1 - appendix) shows the range of themes within the categories and what topics arose. The arms of the map show the breadth of views expressed.

### The meaning of spiritual care

As previously noted, spiritual care is a poorly defined concept, meaning different things to different people. The participants were asked what it meant to them. Although religious support was a key element, it was not the only thing mentioned. Participants communicated a wider view of spirituality involving pastoral care and a holistic view of healthcare in which spiritual care has an important role.

*‘I think it’s not just a religious thing, is it really, the pastoral side of it is more to talk to people and to help them’ (67)*

Most participants described themselves as religious. Their spirituality was interwoven with their religion. For them, spiritual care needed to incorporate religion. It might be delivered by:

*‘Someone who is from a religious order who prays with you and helps you with spiritual questions and helps you understand your faith better.’ (20)*

Some described the chaplains’ work as a key part of their healthcare.

*‘[spiritual care’s] very very important for mental health; sometimes it’s the only thing that seems, that can maybe get through to someone. It’s a different sort of level of understanding, that goes beyond words that goes beyond, something you can touch, it goes beyond all that and I do believe in the power of Grace. I do believe in the Almighty God and I do believe that Jesus was the best healer that this world had ever known.’ (21)*

### The benefits of the SPC department

The participants listed many SPC services, ranging from providing formal religious services, to the more pastoral ‘*having someone to talk to*’, as helpful. Table 3 shows the specific SPC services participants valued (at least two participants mentioned each service in the table).

**Table 3:** Helpful services provided by SPC staff

Religious provision	Pastoral provision
Formal religious service	Listening
Prayer	The social side of religious services
Spiritual advice/guidance	Providing an emotional connection
Holy Communion*	Providing hope/self-worth
Confession^	A critical friend
Normalising faith	A bridge between community and ward
	‘Tending the good in someone’

*Holy Communion, shared between Christians, involves breaking bread and wine to commemorate Jesus’ life, death, and resurrection. It is usually a communal ceremony

^Confession, a mainly Roman Catholic practice, involves sharing perceived wrongdoings with a priest in the anticipation of divine forgiveness

Health professionals are generally less religious than service users [23]. This has caused some service users difficulty in expressing religious ideas for fear of the being considered psychotic [24]. The presence of a chaplain on the ward was seen as ‘normalising faith’, meaning faith was seen as a normal occurrence. This gave service users confidence to speak about faith or look for support in accessing it without feeling their request would be considered pathological.

Pentecostal participants and those from secure services (from several Christian denominations) emphasised the social side of services and the fellowship that it provided. Feeling part of a community, valued, and loved was important.

*‘Having fellowship is important’ (21)*

Participants found involvement in planning and delivering formal religious services helpful as was the social side of services. Pentecostal and Anglican participants suggested Bible study groups. The Roman Catholics highlighted strength received from God to help in recovery. The Born-Again Christian participant spoke of the benefit of accessing an evangelical faith healer, though no one else mentioned it. This may be specific to certain Evangelical denominations and not a regular request.

One frequent comment was that chaplains helped service users find hope. This spiritual resilience was important to many.

*‘I find it helps me, you know, it helps me no end, you know in all sorts of ways. Sometimes I might have been having a particularly you know, particularly bad week, overwhelming, Sister [---] comes and I have communion and I sit and reflect and you know, it means so much to me and it brings me back up’ (32)*

### The role of religion

Many noted access to church or chaplains helped them in various ways included feeling at one with God, expecting God would directly intervene or providing hope and strength.

*‘It wasn’t like I needed to speak to the priest or anything special. It was just to be part of that Christian service, and have the chance to pray and things and just feel that I was part of that service and part of prayer opportunity and to sort of I don’t know maybe feel I was squaring something with God or something. Because I felt angry about the situation and somehow it seemed to work for me, I felt there was a certain resolution in my own mind about what had happened by just being there.’ (68)*

Some participants understood God as the primary agent of healing and source of hope for recovery. Supporting (and sometimes moderating) this belief could benefit therapeutic relationships with other professions. ‘*I think healing is a miracle from God’* (21)

For most, their faith provided strength, hope, and self-worth rather than God providing any direct intervention. These are key aspects of resilience and mental well-being, essential for mental health recovery.

Participants wanted religious activities one might find in an ordinary parish, including prayer, confession, communion, and Bible study. Christian and atheist participants showed clear respect for other religions.

*‘I just treat every religion the same’ (33)*

Although participants respected other faith leaders, they preferred a chaplain of their own faith. For some Roman Catholics and the Born-Again Christian, denomination was important.

Even in Liverpool, which is more religious than the United Kingdom average, some people reported stigma associated with being religious.

*‘You don’t want people laughing in front of us while we’re praying and that.’ (23)*

*‘I find it difficult when people put me down for my faith. Again going back to ‘oh are you going God bothering’ and people don’t understand me I think.’ (31)*

### Qualities of a ‘good’ chaplain

All participants felt ‘good’ staff, regardless of their profession, were distinguished by human qualities such as listening and compassion rather than by technical skill. ‘Good’ staff were empathetic and kind. Participants felt ‘bad’ staff were those they saw as overworked. They stressed it was the hospital management’s responsibility to prevent overworking as it impacted negatively on patients. This mirrors research showing a link between burnout in staff and lower levels of patient satisfaction [25]. Table 4 shows some of the characteristics participants said they looked for in a good chaplain. The human or pastoral qualities were those you would look for in any health professional, the ability to represent God distinguishing the chaplain.

**Table 4:** Participants’ views on what made a ‘good chaplain’

Human qualities	‘Man of God’
Non-judgemental	Be a ‘man of God’
Honest	Church leader/spiritual training (whether this necessitates ordination varies)
Approachable	
Trustworthy	Walk with God
Genuine	Have a prayerful life
Kind	Have a genuine relationship with God
Friendly	Bring the word of God
Confidence	The ability to represent multiple faiths
Empathetic	Channel the Grace of God
Critical friend	
Have time	
Good communication	
Life experience	
Down to Earth	
Knowledge of the mental health system	

Younger participants tended to focus more on the need for the chaplain to be an ordained minister. Older participants were more concerned that the chaplain had life experience.

The list of human qualities was similar across all demographics [26]. Roman Catholics more often reported wanting an ordained priest than other groups. Participants differed around the importance attached to ordination. Many said they were unconcerned about a chaplain’s qualifications, preferring life experience as a quality, but then listed services often requiring a highly-trained individual. These included: Mass, confession, teaching scripture, and the meaning of the Bible, linking scripture with modern events, communion, and church services.

### Who talks to chaplains and when

The general feeling was that everyone, regardless of faith background, would need pastoral care. Although people of other faiths were well respected, people of no faith were generally considered to be ‘*unawakened*’ and in need of conversion. Many respondents felt bringing non-believers into the fold was one of a chaplain’s roles.

*‘It would be nice to say the non-religious to try to get them to change their minds and that there is a God, because those are the ones that need the help, not the religious ones.’ (19)*

Whilst NHS staff are prohibited from evangelising, it was a commonly expressed wish from the service user participants.

There was a feeling that SPC services do not reach widely enough and chaplains should serve carers and community patients. Some suggested chaplains acting as a bridge between community and ward [27, 28].

*‘I would say if they have been a member of a church before, that might not be too difficult but if they have never been there needs to be some sort of cooperation between the chaplaincy and the people ….who are the pastors in the community’ (21)*

On the ward it was acknowledged that people would benefit from seeing a chaplain at different times. This links to themes about advertising so patients know what services are available and how to access them when they need them. Several participants suggested need could be unanticipated and wanted an on-call chaplain. They recommended a regular presence on the ward so patients could expect someone coming round at a certain time on a certain day. This would mean that they could make sure they did not go out on leave and miss the chaplains. This was especially important on wards without an onsite chaplain.

Secure service participants suggested following the prison model, where someone admitted would see a chaplain within 24 hours. This was valued because it welcomes the patient, lets them know what services are available and shows a friendly face at a distressing time. Waiting until someone can leave the ward, perhaps months or years into their stay, before the chaplains made contact was considered inadequate.

*‘I feel like when new patients arrive in the hospital, someone from the spiritual care should go and see them, straight away. To make them aware that there is a church service going on every week and they, what it’s about and making people feel welcome and accepted into the church.’ (19)*

The participants were generally positive about current SPC services; however, they had many suggestions for improvements. Universally, participants felt that chaplains were under time pressure. They wanted to see chaplains more often and have more available. They wanted an increase in services (especially on Sundays), Bible study groups, and hymn practices, as well as more informal association.

*‘like a Bible study but prepare for the Sunday coming so that the patients together with the staff are designing the service’ (62)*

### Chaplains and the multidisciplinary team

Although the interview questions asked specifically about the SPC department, participants also made many comments about wider issues. Common statements considered differences between chaplains and other professionals and chaplains’ role in the multidisciplinary team. Most reported a good relationship with the nursing staff but found chaplains easier to talk to.

*‘But the psychologist inevitably has an alternate agenda… Yeah the chaplain just listens and doesn’t necessarily have an opinion on it or an ulterior motive.’* (95)

There was a sense other professionals spent much time monitoring service users, trying to find out about them, or seeking to change them. Chaplains were seen differently, as having no agenda or goal other than listening.

Chaplains’ integration into the multidisciplinary team (including access to patient notes, being part of ward rounds and care planning activities) divided opinion around personal preference rather than demographics. Those in favour suggested it would normalise faith and facilitate accessing SPC. They said it would improve communication between different services. Those wanting a separate SPC felt it would make talking to chaplains harder without the ‘confidentiality of the confessional’. There is a sense that chaplains offer a fundamentally different type of service to other hospital staff.

*‘I prefer to keep my spiritual needs to one side and my nursing team to another side because it’s a different approach it is a different sort of mind set’* (31)

Those mentioning confidentiality accepted chaplains were NHS staff and had to pass on risk information but preferred they didn’t pass on anything else.

Some religious participants were uninterested in SPC religious services, preferring the local church on a Sunday morning rather than the hospital chapel.

*‘I’d like to go to Mass on a weekend’* (66)

Most could not do this as they would need staff escorts. Most wards run on fewer staff at the weekend, meaning escorted leave was harder to grant.

*‘There is a Catholic Church only round the corner only they won’t let me go there, they won’t take people who want to go to church because they haven’t got the staff.’* (67)

This lack of provision was criticised. One participant felt ward staffing should be highest on a Sunday morning to allow large scale church attendance.

### Grounded theory

**Fig. 2:**
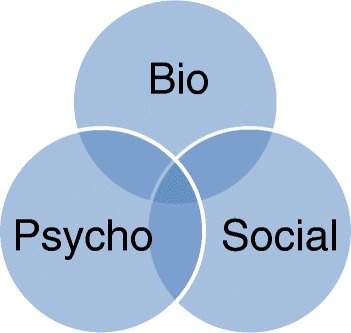
A representation of the bio-psycho-social model

Most NHS mental health services use the bio-psycho-social model [29] (Fig. 2). This revolves around separate but interacting biological, psychological, and social dimensions of health, illness, and well-being. NHS treatments (including social care) focus on one or more of these dimensions.

**Fig. 3:**
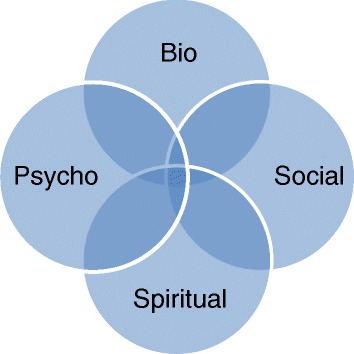
The traditional view of the bio-psycho-social-spiritual model

Some participants felt this model is missing a key component – the spiritual. They specified why spirituality was important. One participant spoke of how her belief in God had helped motivate her to work with a psychotherapist. Another said she disliked taking tablets but felt God had revealed this knowledge so it was okay to take them. These explanations and motivations may not suit everyone but, for these service users, they were key to engaging with treatment. Others spoke of the importance of fellowship, feeling loved and being part of a community, and how that helped combat the isolation of mental illness. Others talked of the peace and calming nature of prayer and attending religious services. All these aspects are clearly important in mental health recovery [10] but not easily contained within the bio-psycho-social model. Adding spirituality to the bio-psycho-social model has been suggested before [30] although usually as an additional but equal element, represented by a fourth identical circle in the diagram (Fig. 3). This reinterpretation identifies four distinct but interacting dimensions of a person’s well-being, none of which can be removed from the whole [30].

**Fig. 4:**
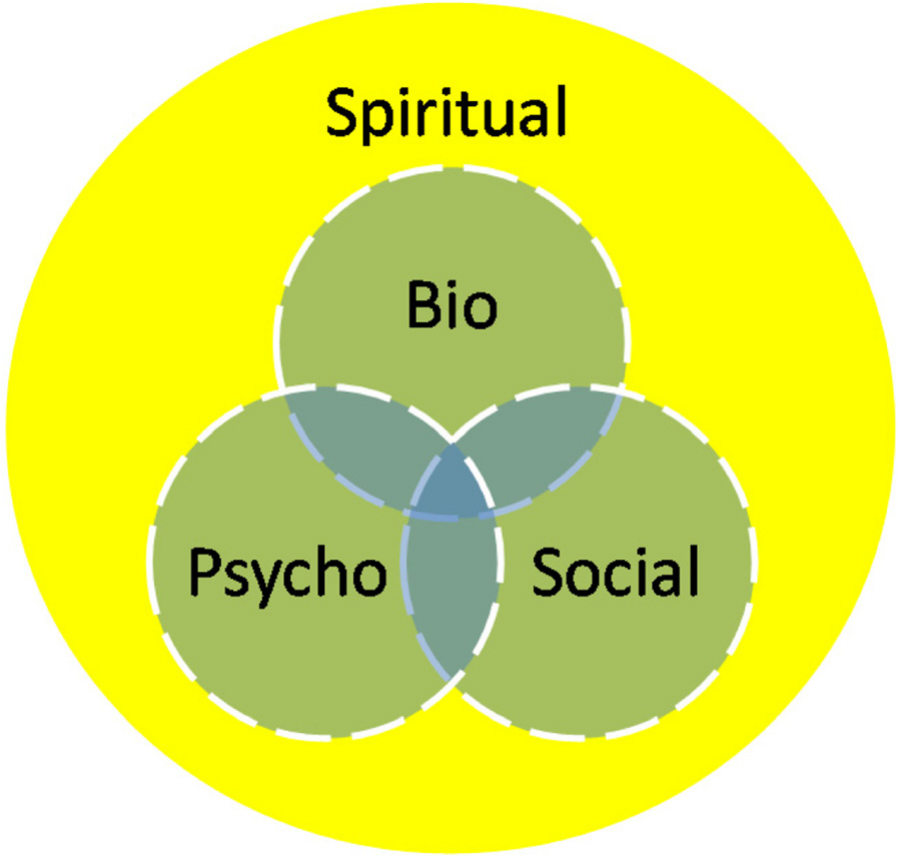
A revised bio-psycho-social-spiritual model of care

This study’s findings suggest spirituality interacts with the other dimensions and yet also transcends them. A revised model is therefore proposed (Fig. 4) with the participants’ views more accurately representing the place of spirituality in healthcare. They identified it as crucial to engaging the other dimensions. For example, if services failed to respect the beliefs of the participant who took medication because she saw it as God-given, she may have refused it. Engaging in psychotherapy is often a very challenging experience for service users. If they gain motivation by believing God is helping them, it should not be ignored as a source of strength and resilience. A service user without hope has a poor prognosis and many people draw hope from religious belief. Religious communities, formal and informal, within NHS services and in the wider community can provide a sense of belonging, of fellowship, and being part of a greater whole.

The model can use a lighter colour where the spiritual dimension is less salient in a person’s experience. For some service users, the yellow (spiritual) will be pale, perhaps transparent, and insignificant for them either in terms of providing resilience or meeting needs. For most of our participants, it was their motivation. Ignoring this motivation will alienate them and fail to engage them in their care. The dotted lines reinforce our finding that all concepts interact and affect each other.

## Discussion

This study’s findings are generated by the confluence of the researchers’ commitment to co-production (of both SPC and research) and a constructivist grounded theory methodology. They force a rethink of the bio-psycho-social model of mental health, suggesting modification to recognise spirituality and spiritual care. Seeing the spiritual as a wider dimension interacting with some, all, or none, of the bio-psycho-social dimensions explains discrepancies. It explains why our participants see spiritual care as an essential part of care whereas others disregard it.

If the bio-psycho-social model is considered in more fluid terms, as the interaction of influences and social processes, a different picture emerges. Spirituality may then be considered in significant part to be a mechanism used by service users to retain their sense of self in response to being treated according to the bio-psycho-social model. It may be a defence against perceived (or actual) totalising influences, whether directly attributable to treatment or broader aspects of institutionalisation. For most of the participants, it expressed itself in overtly religious terms but this need not necessarily be the case. This interpretation of spirituality resembles understandings of patient experience [31]. It is not intrinsically hostile to treatment (the example of the woman who saw medication as God-given was very supportive). It warrants sensitive exploration to build therapeutic relationship and address anxieties. Ignoring spirituality is likely to compromise effectiveness. The spiritual, thus understood, helps make sense of some instances of frustration and aggression. The authors’ opinion is that health service professionals should engage with this element regardless of whether a person presents as spiritual, religious, or otherwise. Differences need to be acknowledged, respected, and considered when planning care [32, 33]. Whilst advocating this understanding, it is also important not to subordinate all spiritual expression to a response to treatment or institutionalisation. Many service users ordinarily engage in spiritual practices that they would value being able to continue when hospitalised.

This study aimed to learn what SPC users wanted from the service. All participants valued the SPC department and felt that seeing a chaplain had helped their recovery. Most could see ways of improving SPC; none found it unhelpful. Further work on how chaplains can best support the spiritual needs of those without faith is required [34]. Some participants in this study felt one role of chaplains should be to convert those with no faith, however, support, not proselytising, is the role of NHS chaplains.

The role of the chaplain included a variety of human or pastoral roles as well as a faith representation. It was viewed as clearly differentiated from the role of other professionals, though how closely chaplains should work with those other professionals was contested. Spiritual care services appear to be more aligned with the recovery processes of connectedness, hope, identity, meaning in life and empowerment (CHIME) than in traditional healthcare approaches of the medical model, although it can certainly work with those approaches where necessary.

Our six categories reflect other research on the domains of spiritual care. Burkhart and Hogan (2008) ran focus groups with American nurses to study their role in spiritual care. Their grounded theory research reported nurses saying that spiritual care came in the three categories: promoting patient self-reflection, promoting connectedness between the patient and the family and promoting connectedness between the patient and God [35]. The self-reflection theme closely resembles the pastoral care that chaplains provide; listening and philosophical discussion. Promoting connectedness between the patient and God closely resembles the religious aspects that our participants expected from chaplains. There was little mention in our interviews about promoting connectedness between patient and family. Some people suggested chaplains for carers but distinct from the patient.

The USA is a more overtly religious society than the UK, with average church attendance around 50 % [36] as opposed to 15 % in the UK [37]. Reflecting these cultural differences, Burkhart and Hogan’s nurses regularly prayed with their patients, though only if initiated by the patient. As in the UK, the American nurses highlighted that their nurse training did not prepare them for delivering spiritual care [35].

Koslander and Arvidsson (2007), again using grounded theory, explored patient perspectives on spiritual care in Swedish mental health settings. They identified three main categories: (1) it was important to patients that spiritual needs were met, (2) patients felt it was up to them to be proactive in making sure they received spiritual care, and (3) patients lacked confidence in talking to nurses about spiritual care [38]. This generally reflected the responses from participants in our study although Koslander and Arvidsson made no reference to chaplains. The Swedish patients were keen to talk to their nurses about spirituality. Though our participants were less likely to want this, they still thought nurses should know about the issue, be willing to discuss spiritual care, and know about available SPC services.

The present study’s findings are also consistent with Rosmarin and colleagues’ (2015) observation that it is important to offer suitable spiritual care to mental health service users. Simply recording a service users’ religious affiliation is inadequate [39]. Rosmarin used a survey to gauge service user attitudes to spiritual care in a Massachusetts hospital. Although mainly asking about spiritually integrated psychotherapy rather than SPC, well over half of the respondents were keen to have a spiritual dimension to their care [40]. Walsh reported that proper consideration of the spiritual dimension could not be presumed upon in the NHS [39].

Co-producing the research was felt to be a valuable experience with deep respect developing between the members of the Panel. Involving people with lived experience of using mental health services from the outset and sharing experience throughout the research cycle significantly improved the fidelity of the research and facilitated recruitment into the study. Overall, most participants revealed profound insight into their experience and many of their stories were deeply moving. Being part of this research project has felt a privilege in every way and the researchers would hope the expectation would be that all future UK-based research would be co-produced be default.

### Limitations of the study

This has been a small qualitative study in a more religious and more Christian than average part of the UK. Participants’ opinions may not represent the UK as a whole, or mental health service users in other countries. Nonetheless, they are helpful for service design in this part of the country; and the service user view that spirituality is an important part of their holistic treatment is supported by studies from other countries [40, 41].

Almost all the participants were inpatients from open acute or secure wards. Older adults with dementia and smaller services (including brain injury, drug and alcohol services, and learning disability) were excluded. Our sample was disproportionately male and older than the average inpatient. Although everyone on the included wards was invited, those without interest in SPC may have declined to be interviewed.

Both the Panel and participants reflected a wide spread of demographics and educational attainment but the findings’ generalisability may be questioned at two levels. The first concerns the details regarding aspects of SPC that were valued. Different findings might have been generated elsewhere or at different times or by different researchers. More challenging is the implication that co-production would be desirable world-wide. There is real risk of dogmatically imposing a seemingly benign Eurocentric model on cultures where it may be inappropriate or actually harmful. Authentic co-production would allow for genuine consultation but is predicated on profound respect and considerable skill in communication. Superficial or coercive pseudo co-production would not only be paternalistic but could potentially be more damaging than its displaced alternative.

The initial coding was undertaken by EW who is not theologically trained. Although the coding frame was cross-checked and discussed in depth with researchers who are, it may have been done differently by a chaplain.

## Conclusions

In common with other recent studies [35, 38, 40], this study shows mental health service users are keen to have spiritual and religious elements to their care. Many regard this as essential to the healing process. NHS services should consider a bio-psycho-social-spiritual model in their aim to provide holistic, patient centred care to their patients. Co-producing the research has proved invaluable.

## Abbreviations

NHS, National Health Service, SPC, Spiritual and Pastoral Care
